# Pseudo-Anaphylactic Reactions to Pfizer BNT162b2 Vaccine: Report of 3 Cases of Anaphylaxis Post Pfizer BNT162b2 Vaccination

**DOI:** 10.3390/vaccines9090974

**Published:** 2021-08-31

**Authors:** Xin Rong Lim, Bernard Pui Leung, Carol Yee Leng Ng, Justina Wei Lynn Tan, Grace Yin Lai Chan, Chien Mei Loh, Gwendolyn Li Xuan Tan, Valerie Hui Hian Goh, Lok To Wong, Chong Rui Chua, Sze Chin Tan, Samuel Shang Ming Lee, Hwee Siew Howe, Bernard Yu Hor Thong, Khai Pang Leong

**Affiliations:** 1 Department of Rheumatology, Allergy and Immunology, Tan Tock Seng Hospital, 11 Jalan Tan Tock Seng, Singapore 308433, Singapore; bernard.p.leung@gmail.com (B.P.L.); justina_tan@ttsh.com.sg (J.W.L.T.); grace_yl_chan@ttsh.com.sg (G.Y.L.C.); sze_chin_tan@ttsh.com.sg (S.C.T.); samuel_lee@ttsh.com.sg (S.S.M.L.); Howe_hwee_siew@ttsh.com.sg (H.S.H.); bernard_thong@ttsh.com.sg (B.Y.H.T.); khai_pang_leong@ttsh.com.sg (K.P.L.); 2 Health and Social Sciences, Singapore Institute of Technology, Singapore 138683, Singapore; 3 Clinical Immunology Laboratory, Tan Tock Seng Hospital, Singapore 308433, Singapore; carol_ng@ttsh.com.sg (C.Y.L.N.); chien_mei_loh@ttsh.com.sg (C.M.L.); Gwendolyn_TAN@ttsh.com.sg (G.L.X.T.); Hui_Hian_Valerie_GOH@ttsh.com.sg (V.H.H.G.); Lok_To_WONG@ttsh.com.sg (L.T.W.); Chong_Rui_CHUA@ttsh.com.sg (C.R.C.)

**Keywords:** COVID-19, vaccine, anaphylaxis, antibodies, cytokines

## Abstract

Anaphylactic reactions were observed after Singapore’s national coronavirus disease 2019 (COVID-19) vaccination programme started in December 2020. We report the clinical and laboratory features of three patients in our institution who developed anaphylactic reactions after receiving the Pifzer BNT162b2 vaccine. IgM and IgG antibodies, but not IgE antibodies to the Pfizer BNT162b2 vaccine, were detected in all subjects. Similarly, mild to high elevated levels of anti-polyethylene glycol (PEG) IgG (1035–19709 U/mL, vs. vaccine-naive < 265 U/mL, vaccine-tolerant < 785 U/mL) and IgM (1682–5310 U/mL, vs. vaccine-naive < 1011 U/mL, vaccine-tolerant < 1007 U/mL) were detected in two out of three patients via commercial ELISA. High levels of serum anaphylatoxin C3a (79.0 ± 6.3 μg/mL, mean ± SD, vs. normal < 10 μg/mL) were observed in all three patients during the acute phase of the reaction, while tryptase levels, a marker of mast cell activation, were not elevated. Finally, one patient with the highest levels of anti-PEG IgG, IgM, and anti-Pfizer BNT162b2 IgG and IgM exhibited an enhanced Th2 cytokine serum profile during an acute reaction, with high levels of IL-4 (45.7 pg/mL, vs. vaccine-naive/tolerant < 2.30 pg/mL), IL-33 (86.4 pg/mL, vs. vaccine-naive/tolerant < 5.51 pg/mL) and IL-10 (22.9 pg/mL, vs. vaccine-naive/tolerant < 12.49 pg/mL) diminishing over time following corticosteroid treatment. Taken together, we propose these cases of anaphylaxis described are driven by a complement activation-related pseudoallergy (CAPRA), rather than classical IgE-mediated mechanisms.

## 1. Introduction

The United States (US) Food and Drug Administration (FDA) granted emergency use authorization of the Pfizer BNT162b2 vaccine on 11 December 2020. On 6 January 2021, the US Centers for Disease Control (CDC) announced that there had been 21 cases of anaphylaxis resulting from the Pfizer BNT162b2 vaccine in the period 14–23 December [1]. In this period, 1,893,360 first doses of the vaccine were administered, with a reaction rate of 11.1 cases per million doses. Most of the reactions (71%) occurred within 15 min of vaccination. A total of 17 of the 21 people had a documented history of allergies or allergic reactions and seven had a history of anaphylaxis.

Singapore’s Health Sciences Authority (HSA) granted interim authorisation for the Pfizer BNT162b2 vaccine under the Pandemic Special Access Route (PSAR) on 14 December 2020 [2] and the Moderna mRNA-1273 vaccine on 3 February 2021 [3]. As of 18 April 2021, amongst 2,213,888 doses of vaccine administered, there were 20 cases of anaphylaxis reported with the Pfizer BNT162b2 and Moderna mRNA-1273 vaccines [4]. This is similar to the incidence rates reported overseas of around 0.5 to 2 per 100,000 doses administered. Our institution commenced Pfizer BNT162b2 vaccination on 30 December 2020. Within the first 2 months of vaccine inoculation, there were five cases of anaphylaxis among healthcare workers in our hospital, with three who consented to our study and fulfilled the Brighton Collaboration Anaphylaxis Working Group’s case definition [5] (Table 1).

## 2. Methods

### Antibodies to Pfizer BNT162b2 Vaccine, Anti-PEG IgG and IgM, Total Serum Tryptase, C3a and Cytokine ELISAs

Serum anti-Pfizer BNT162b2 vaccine antibodies, from here on referred to as anti-BNT162b2 IgM, IgG, and IgE levels were measured by ELISA. Briefly, 96-well plates (Nunc-Immuno Maxisorp, ThermoFisher, Waltham, MA, USA) were coated with 1:25 dilution of BNT162b2 vaccine in 0.1 M NaH_2_CO_3_ (pH = 8.6) overnight at 4 °C. Wells were washed twice in PBS/0.02% n-dodecyl-b-D-maltopyranoside (DDM, Avanti Polar Lipids, Inc., Alabaster, AL, USA) and blocked in a sample buffer comprising of 10% bovine calf serum in PBS (Gibco, ThermoFisher Waltham, MA, USA) in PBS/0.02% DDM for 1 h at room temperature (RT). Serum samples were diluted 1:20 in the sample buffer and incubated in the wells for 1 h at RT. Bound antibodies were detected with affinity purified goat anti-human IgM (Sigma-Aldrich, Merck KGaA, Germany), HRP-conjugated anti-human IgG (BD Pharmingen, San Diego, CA, USA), biotin-conjugated anti-human IgE (BD Pharmingen, San Diego, CA, USA), or mouse anti-human IgE (Sigma-Aldrich, Merck KGaA, Germany) in sample buffer for 1 h after wells were washed four times in PBS/0.02% DMM. Wells were washed four times in PBS/0.02% DMM followed by HRP-conjugated anti-goat antibody (Sigma-Aldrich, for IgM detection, Merck KGaA, Germany), HRP-conjugated anti-mouse IgG (BD Pharmingen, for IgE, San Diego, CA, USA), or avidin perioxidase (Sigma-Aldrich, for IgE, Merck KGaA, Germany) diluted in the sample buffer for 1 h at RT. Finally, wells were washed 6 times in PBS/0.02% DDM before being developed with tetramethylbenzidine substrate (Sigma-Aldrich, Merck KGaA, Germany) according to the manufacturer’s instructions. The lowest reliable detection limit was 2.5 standard deviations (SD) from blank. The standard curve was derived by pooling the sera of three BNT162b2 vaccine recipients with the highest optical density of serum IgG or IgM levels with the highest value defined as 200 arbitrary unit (AU/mL). These three vaccine recipients comprise two individuals who developed anaphylaxis post BNT162b2 vaccine and one individual who completed 2 doses of BNT162b2 vaccine with no reactions. Data on IgE were presented as OD values, with all measurements less than 2.5 SDs from blank and considered to be negative. Human anti-PEG-IgE (Hu 6.3 from Academia Sinica, Taipei, Taiwan) served as the positive controls and were detectable at 5 and 200 ng/mL. Given the presence of antigen-specific IgG in our samples, these antibodies may interfere with the binding of the same epitopes for antigen-specific IgE, thereby rendering low-sensitivity in our IgE assays. To this end we have performed the removal of IgG antibodies by employing an IgG/RF stripper (Bio-Rad, Hercules, CA, USA) comprising of sheep anti-serum monospecific to human IgG according to the manufacturer’s recommendations, prior to IgE measurements, as well as an additional IgG ELISA to confirm the level of depletion. We found anti-BNT162b2 IgE levels remained below detection post IgG removal, and our anti-BNT162b2 IgG confirmed the depletion by converting previously positive samples to a negative signal.

By employing the whole BNT162b2 vaccine as the capture antigen, these assays allowed for the measurement of all immunogenic epitopes that can potentially trigger an IgG, IgM, or IgE response. For validation, a spiking recovery experiment was performed using patient samples from a range of autoimmune diseases include systemic lupus erythematosus (SLE) with auto-antibodies to ensure a lack of non-specific binding. Briefly, SLE and other autoimmune disease serum samples (*n* = 10) were spiked with 100 AU/mL of anti-BNT162b2 IgM or IgG. An anti-BNT162b2 IgM or IgG ELISA was then performed as described above and recovery was determined, mean recovery rate: anti-BNT162b2 IgM 104 ± 9.2% (mean ± SD, range 89–117%), anti-BNT162b2 IgG 107 ± 10.6% (mean ± SD, range 89–121%).

Anti-PEG IgG and anti-PEG IgM were measured using life diagnostics (West Chester, PA, USA), serum complement 3a (C3a) level was measured using the (RayBio human C3 alpha, Peachtree Corners, GA, USA), and serum cytokines were assayed by commercial ELISAs according to the manufacturers’ recommendations (BD Pharmingen (San Diego, CA, USA) or ThermoFisher (Waltham, MA, USA)). The lower limit of detection for PEG IgG (0.78 U/mL) and IgM (3.12 U/mL), C3a (2.5 μg/mL); IL4 < 2 pg/mL, IL-6 < 2 pg/mL, IL-10 < 5 pg/mL, IL-33 < 5 pg/mL, MCP < 168 pg/mL, TNF < 3 pg/mL, sTNFRI < 4500 pg/mL. The total tryptase level was measured with the UniCAP-Tryptase fluoroimmunoassay (Phadia, Thermo Fisher Scientific, Uppsala, Sweden) in accordance with the manufacturer’s instructions.

The study was approved by the institutional review board (National Healthcare Group-Domain Specific Review Board Reference Number: 2021/00174) and informed written consent was obtained from each participant. Informed consent was also obtained from six individuals who had not been vaccinated and six individuals who received 2 doses of the Pfizer BNT162b2 vaccine without hypersensitivity reactions 4 to 5 weeks post vaccination. They constituted the vaccine-naive group and vaccine-tolerant group, respectively.

## 3. Case Presentation and Results

Patient 1, a 42-year-old male, developed flushing, periorbital edema, globus sensation, and wheezing 30 min after the second dose of the Pfizer BNT162b2 vaccine (Table 1). He has a history of poorly controlled asthma and urticaria to etoricoxib. He had developed periorbital edema 3 days after the first dose of the Pfizer BNT162b2 vaccine and assumed it was an unrelated event. Patient 1 was managed with two doses of intramuscular (IM) adrenaline and monitored in the high dependency unit. He was hospitalized for 4 days and received 9 days (2 days intravenous (IV), 7 days oral) of moderate-to-high dose corticosteriods in total.

Patient 2, a 32-year-old female, developed erythema, flushing, breathlessness, globus sensation, and wheezing 30 min after the first dose of the Pfizer BNT162b2 vaccine. She has a history of well controlled asthma and mild, intermittent allergic rhinitis. After the initial treatment (Table 1), the symptoms recurred at 8 and 27 h post-vaccination, requiring repeated doses of IM adrenaline. She was hospitalized for 4 days in total and received regular IV hydrocortisone and diphenhydramine throughout the admission.

Patient 3, a 40-year-old female, developed generalised urticaria, periorbital edema, globus sensation, and breathlessness 20 min after receiving the second dose of the Pfizer BNT162b2 vaccine. She has a history of mild, intermittent chronic rhinosinusitis, without asthma or a history of non-steroidal anti-inflammatory drug hypersensitivity. She reported numbness of her left forearm 20 min after the first dose of the Pfizer BNT162b2 vaccine which resolved after 2 days. After initial treatment with IV hydrocortisone and diphenhydramine, she was discharged after 2 days but returned the same evening complaining of the recurrence of periorbital edema and globus sensation. She was admitted for another 2 days and required 7 days of moderate-to-high-dose corticosteriods.

Table 1 summarises the clinical details and laboratory findings of patients 1 to 3, all with a history of allergy. Blood samples from these three patients were collected during the acute reaction, and 4 to 5 weeks post-reaction. Table 2 demonstrates the additional laboratory findings of these patients. IgG and IgM antibodies to PEG in patients 2 and 3 were elevated, in particular anti-PEG IgG versus vaccine-naive (anti-PEG IgG 265 ± 37 AU/mL, anti-PEG IgM 1011 ± 224 AU/mL, mean ± SD, *n* = 6) and vaccine-tolerant subjects (anti-PEG IgG 785 ± 483 AU/mL, anti-PEG IgM 1007 ± 459 AU/mL, mean ± SD, *n* = 6). Similarly, anti-BNT162b2 IgG and IgM antibodies were detected in all three patients, with Patient 2 displaying high levels of anti-BNT162b2 IgG and Patient 3 displaying high levels of anti-BNT162b2 IgG and IgM versus vaccine-naive (anti-BNT162b2 IgG 37.0 ± 17.4 AU/mL, anti-BNT162b2 IgM 31.6 ± 13.0 AU/mL, mean ± SD, *n* = 6) and vaccine-tolerant subjects (anti-BNT162b2 IgG 51.2 ± 29.3 AU/mL, anti-BNT162b2 IgM 23.6 ± 18.3 AU/mL, mean ± SD, *n* = 6). Importantly, none had an elevated tryptase level nor detectable IgE antibodies to the BNT162b62 vaccine. Anaphylatoxin C3a and cytokine data suggested two distinctive mechanisms. Patients 1 and 2 had serum C3a levels exceeding 80 μg/mL (normal < 10 μg/mL) during the acute reaction while allergy-related cytokines including IL-4, IL-10, and IL-33 were not raised. In contrast, Patient 3 had elevated levels of IgG and IgM to both PEG and the Pfizer BNT162b2 vaccine, and high levels of T-helper 2 (Th2) cytokines including IL-4 IL-33, IL-10, TNF Receptor I (TNFRI), and MCP-1, cytokines that are associated with severe anaphylactic reactions [6]. Blood samples of all three patients 4 to 5 weeks post-anaphylaxis showed a reduction in C3a and the majority of the cytokine levels (Table 1 and Table 2). The anti-BNT162b2 assays we employed here detect all potential epitopes that can trigger either an IgG or IgM response, and do not aim to differentiate individual components within the vaccine such as PEG or mRNA encoding the COVID-19 spike protein.

## 4. Discussion

The Pfizer-BioNTech COVID-19 vaccine (BNT162b2) contains modified RNA encoding the coronavirus spike protein encased by a polymer consisting of (4-hydroxybutyl)azanediyl)bis(hexane-6,1-diyl)bis (ALC-3015), (2-hexyldecanoate),2-[(polyethylene glycol)-2000]-N,N-ditetradecylacetamide (ALC-0159), 1,2-distearoyl-snglycero-3-phosphocholine (DPSC), and cholesterol. This vaccine contains several excipients and lipids, and currently PEG-2000 is one of the excipients with recognised allergenic potential [7]. Clinical immunologists and allergists have postulated that such reactions could be due to IgE-mediated mechanisms via anti-PEG IgE or related to pre-existing PEG allergy via anti-PEG IgM or anti-PEG IgG antibodies resulting in a complement activation-related pseudoallergy (CAPRA) [8,9]. Preexisting anti-PEG IgG/IgM triggers complement activation upon exposure to PEGylated liposomes, resulting in the generation of anaphylatoxins C3a and C5a which activates allergic effector cells such as macrophages, basophils, and mast cells [9]. These allergic effector cells release a variety of inflammatory mediators that cause vascular leakage, resulting in the clinical presentation of a pseudoallergy. PEGylated lipid nanoparticles can also directly bind to these allergic effector cells via surface receptors and trigger the secretory response of these cells [10]. While IgE independent pseudoanaphylaxis has been demonstrated in animal models, it is still not clearly demonstrable in human pathology [11].

We observe that the clinical course of these three patients is protracted in nature, lacks hypotension as a feature, has proclivity for symptom recurrence, and requires at least 4–7 days of moderate-to-high-dose corticosteriods. All patients had elevated C3a levels which decreased over time, while tryptase levels remained normal, supporting pseudoallergic reactions as potential mechanisms via a complement activation-related pseudoallergy (CAPRA). The presence of high IgG to PEG and the Pfizer BNT162b2 vaccine in Patient 2 and high IgG/IgM to PEG and the Pfizer BNT162b2 vaccine in Patient 3, and the lack of IgE antibodies suggest that the reactions could be induced by pre-existing anti-PEG antibodies. Patient 1′s reaction could be induced directly by the vaccine lipid nanoparticle activating the complement system, as both antibodies to PEG and the Pfizer BNT162b2 vaccine are low. Anti-BNT162b2 IgG did not rise in all three patients 4 to 5 weeks post-anaphylaxis, and we postulate that this might be related to the corticosteroids that were given as treatment.

Our novel ELISA assay to detect antibodies to the Pfizer BNT162b2 vaccine involves the coating of the Pfizer BNT162b2 vaccine on ELISA plates to allow the binding of antibodies in serum samples against all potential immunogenic epitopes of the vaccine. Further work is required to determine the precise immunogenic epitope of these antibodies directed against the COVID-19 vaccine, whether it is to the PEG component or pegylated lipid nanoparticle of the vaccine. The lack of anti-BNT162b2 IgE and trypase response is intriguing, suggesting a potential mast cell and type 1 hypersensitivity independent event. This was confirmed by employing two different anti-human IgE antibodies in our assays (BD Pharmingen (San Diego, CA, USA) and Sigma-Aldrich (Merck KGaA, Germany)), and additional IgG depletion assays to rule out competitive binding, with all samples < 2.5 standard deviations of optical density from the blank.

Studies have shown that the prevalence of anti-PEG antibodies in healthy populations ranges from 20 to 44% [12]. Population studies are required to determine the prevalance of anti-PEG or anti-BNT162b2 antibodies and to determine a cut-off value that could serve as a diagnostic test to distinguish the at-risk population who will develop anaphylaxis to mRNA COVID-19 vaccines.

## 5. Conclusions

To date, the cause of anaphylaxis post mRNA COVID-19 vaccination remains unclear. Many authors postulate that PEG could be a potential allergen via IgE- and non-IgE-mediated mechanisms [8,9,10]. Further studies are required to dissect the immunological mechanisms of anaphylaxis post COVID-19 mRNA vaccination, with larger cohorts for the prevalance of anti-PEG and/or anti-BNT162b2 antibodies, and to establish a potential cut-off value for COVID-19 mRNA vaccine-related anaphylaxis.

## Figures and Tables

**Table 1:** Clinical details and laboratory findings of patients 1–3 and controls.

Patient	Reaction Onset	Atopy History	Signs and Symptoms	Brighton Level /Vaccine Dose	Treatment	Time of Blood Collection after Onset of Anaphylaxis	Tryptase ng/mL	C3a ug/mL
1	30 min	Asthma, urticaria to etoricoxib	Flushing, periorbital edema, globus sensation, wheezing	1 (Second dose)	IM Adrenaline X 2 doses, IV hydrocortisone, IV diphenhydramine, nebulised salbutamol	3 h	3.2	83.2
						5 weeks	N.A.	58.6
2	30 min	Asthma, allergic rhinitis	Flushing, erythema, breathlessness, globus sensation, wheezing	1 (First dose)	IM adrenaline, IV hydrocortisone, IV diphenhydramine, IV cimetidine, nebulised salbutamol and ipratropium	6 h	2.2	82.1
						41 h	3	81.2
						4 weeks	N.A.	9.0
3	20 min	Chronic rhinosinusitis	Generalised urticaria, periorbital edema, globus sensation, breathlessness	2 (Second dose)	IM adrenaline, IV hydrocortisone and diphenhydramine	6 h	2.3	71.7
						22 h	1.9	28.0
						5 weeks	N.A.	2.5
Vaccine-naive controls (*n* = 6), Mean ± SD						Not Applicable	N.A.	<10
Vaccine-tolerant controls (*n* = 6), Mean ± SD						Not Applicable	N.A.	<10

Normal cut-off as follows: Tryptase < 11.4 ng/mL (according to manufacturer’s instructions) Abbreviations: N.A., Not Available; IM, Intramuscular; IV, Intravenous.

**Table 2:** Immunological findings of patients 1–3 and controls.

Patient	Time of Blood Collection after Onset of Anaphylaxis	PEG IgG U/mL	PEG IgM U/mL	Pfizer C-19 Vaccine IgG AU/mL	Pfizer C-19 Vaccine IgM AU/mL	Pfizer C-19 Vaccine IgE AU/mL	IL-4 pg/mL	IL-6 pg/mL	IL-33 pg/mL	sTNFRI pg/mL	TNF pg/mL	IL-10 pg/mL	MCP1 pg/mL
1	3 h	93	859	15.9	29.6	N.D.	0.32	0.46	4.39	2635.2	0.34	8.97	198.3
	5 weeks	209	1004	14.6	27.6	N.D.	0.14	0.56	3.24	2670.4	0.19	9.08	268.8
2	6 h	1035	1682	101.6	42.5	N.D.	0.14	0.28	4.39	2658.6	0.39	8.45	195.7
	41 h	1021	1560	102.7	40.0	N.D.	0.14	0.97	0.94	2623.5	0.39	9.19	211.4
	4 weeks	1474	2681	107.7	41.7	N.D.	0.14	0.88	0.94	2670.4	0.19	12.30	308.9
3	6 h	13,054	5091	176.1	138.5	N.D.	45.69	2.10	86.36	5450.4	0.84	22.98	439.8
	22 h	19,709	5310	266.4	86.6	N.D.	5.07	0.85	11.49	3745.9	0.19	11.74	305.5
	5 weeks	10,493	3934	124.2	98.6	N.D.	7.72	1.13	18.19	4463.8	0.29	11.48	409.3
Vaccine-naive controls	N.A.	265 ± 37	1011 ± 224	37.0 ± 17.4	31.6 ± 13.0	N.D.	2.30 ± 1.11	1.70 ± 2.30	5.51 ± 2.73	4679.9 ± 1620.7	1.31 ± 2.84	12.49 ± 7.81	312.3 ± 107.5
Vaccine-tolerant controls	4–5 weeks after 2nd dose of Pfizer C-19 vaccine	785 ± 483	1007 ± 459	51.2 ± 29.3	23.6 ± 18.3	N.D.	1.26 ± 0.95	1.00 ± 1.05	4.47 ± 2.90	3492.1 ± 1181.1	1.56 ± 3.41	10.94 ± 2.75	254.08 ± 32.83

N.D., Not Detectable, Pfizer C-19 Vaccine, Pfizer COVID-19 Vaccine; PEG, Polyethylene Glycol, Mean ± SD.

## Data Availability

The data presented in this study are available on request from the corresponding author. The data are not publicly available due to privacy and ethical reasons.
